# Optimization of Ecological Water Supplement Scheme for Improved Suitable Habitat Area for Rare Migratory Birds in Nature Reserves Using Interval-Parameter Fuzzy Two-Stage Stochastic Programming Model

**DOI:** 10.3390/ijerph17207562

**Published:** 2020-10-17

**Authors:** Xianrui Liao, Chong Meng, Zhixing Ren, Wenjin Zhao

**Affiliations:** 1 College of New Energy and Environment, Jilin University, Changchun 130012, China; liaoxr18@mails.jlu.edu.cn; 2 College of Environment, Beijing Normal University, Beijing 102206, China; meng@bnu.edu.cn; 3 College of Forestry, Northeast Forestry University, Harbin 150040, China; renzhixingryy@outlook.com

**Keywords:** rare migrant birds, ecological water replenishment, nature reserve, habitat area, carrying capacity for rare birds, interval-parameter fuzzy two-stage stochastic programming method

## Abstract

The optimization of ecological water supplement scheme in Momoge National Nature Reserve (MNNR), using an interval-parameter two-stage stochastic programming model (IPTSP), still experiences problems with fuzzy uncertainties and the wide scope of the obtained optimization schemes. These two limitations pose a high risk of system failure causing high decision risk for decision-makers and render it difficult to further undertake optimization schemes respectively. Therefore, an interval-parameter fuzzy two-stage stochastic programming (IPFTSP) model derived from an IPTSP model was constructed to address the random variable, the interval uncertainties and the fuzzy uncertainties in the water management system in the present study, to reduce decision risk and narrow down the scope of the optimization schemes. The constructed IPFTSP model was subsequently applied to the optimization of the ecological water supplement scheme of MNNR under different scenarios, to maximize the recovered habitat area and the carrying capacity for rare migratory water birds. As per the results of the IPFTSP model, the recovered habitat areas for rare migratory birds under low, medium and high flood flow scenarios were (14.06, 17.88) × 10^3^, (14.92, 18.96) × 10^3^ and (15.83, 19.43) × 10^3^ ha, respectively, and the target value was (14.60, 18.47) × 10^3^ ha with a fuzzy membership of (0.01, 0.83). Fuzzy membership reflects the possibility level that the model solutions satisfy the target value and the corresponding decision risk. We further observed that the habitat area recovered by the optimization schemes of the IPFTSP model was significantly increased compared to the recommended scheme, and the increases observed were (5.22%, 33.78%), (11.62%, 41.88%) and (18.44%, 45.39%). In addition, the interval widths of the recovered habitat areas in the IPFTSP model were reduced by 17.15%, 17.98% and 23.86%, in comparison to those from the IPTSP model. It was revealed that the IPFTSP model, besides generating the optimal decision schemes under different scenarios for decision-makers to select and providing decision space to adjust the decision schemes, also shortened the decision range, thereby reducing the decision risk and the difficulty of undertaking decision schemes. In addition, the fuzzy membership obtained from the IPFTSP model, reflecting the relationship among the possibility level, the target value, and the decision risk, assists the decision-makers in planning the ecological water supplement scheme with a preference for target value and decision risk.

## 1. Introduction

The wetland ecosystem is one of the most important ecosystems on earth, as it provides necessary ecosystem services such as flood control, climate regulation and habitat provision [1,2,3,4,5,6]. Wetland serves as a stop-over region for rare migratory water birds. Wetland restoration is important for the protection of the suitable habitat of water birds [7], and ecological supplement is an effective and efficient approach for the restoration of the ecological functions of a wetland [8]. Momoge National Nature Reserve (MNNR) is among the most important natural reserves in China. However, the wetland area and the suitable habitat area (water areas of depth between 0–40 cm) for rare migratory water birds in MNNR have been reduced to a great extent, which has led to a sharp decrease in the number of rare migratory water birds visiting MNNR [9]. Currently, an ecological water supplement project is being implemented in the nature reserve by the government. In a previous study, Liao et al. selected 18 lakes in MNNR as the study area and developed an interval-parameter two-stage stochastic programming (IPTSP) model to optimize the ecological water supplement scheme (the scheme of replenishing ecological water to lakes in MNNR) recommended by project plan, which was reported to significantly improve the suitable habitat area for rare migratory water birds in MNNR, compared to the project recommendation scheme [10]. However, besides interval parameters (ecological service value, ecological water demand, etc.) and random variable (flood resources), there are also fuzzy uncertainties (the area of ecological function regions (the 4 types of regions with different ecological functions, including the fish pond, crab pond, reed, and marsh wetland) and the total planning area of lakes) in the water resource planning systems of MNNR, which present risks and challenges in the optimization and implementation of ecological water supplement schemes. Moreover, the scope of optimization of the ecological supplement scheme is quite wide, rendering it difficult to undertake decisions. The IPTSP model is incapable of addressing these problems, which causes decision-makers to face inevitable decision-making risk.

Owing to the complexity of the water resource management and planning systems, there exist complex information and several uncertainties, including the random variables such as flood resource and surface and groundwater availability [11,12], the interval parameters such as economic benefits [13], and the fuzzy variables such as seasonal flow [14]. The accurate quantification of these uncertain parameters and variables is difficult, presenting risks and difficulties in the scientific management and utilization of water resources [15,16]. Currently, for addressing uncertainties, the following three uncertainty programming methods are used widely: interval linear programming (ILP), stochastic mathematical programming (SMP), and fuzzy mathematical programming (FMP). 

ILP may represent parameters and results as interval values to address interval uncertainties [17]. As an example, Fu et al. developed an interval two-stage stochastic robust programming model for the optimization of irrigation water allocation in Jiamusi city, China [18]. SMP is capable of effectively handling the random variables expressed as known probability density functions [19], and it includes chance-constrained programming (CCP) and two-stage stochastic programming (TSP). As an example, Cai et al. proposed a double-sided chance-constrained method for the optimization of water allocation for higher ecological benefits [20]. TSP is capable of modifying the predetermined objectives and generating corresponding decisions after the occurrence of uncertain events and is also convenient when combined with other uncertainty programming methods in order to address multiple uncertainties simultaneously [21]. As an instance, Ji et al. developed an inexact two-stage stochastic programming model for the optimization of regional water resource allocation [22]. However, the wide decision range and a high decision risk of the optimization schemes of the IPTSP model create difficulties for managers in undertaking decisions. In addition, the solutions from the IPTSP model do not provide the possibility of satisfying the target value, which is not conducive to tradeoff the relationship between the target value and decision risk. These problems could be addressed by using FMP, which adopts the theory of fuzzy set to handle fuzzy uncertainty and is applied widely for planning and managing water resources because of the small amount of data required in this method [23,24,25]. As an example, in their study, Maqsood et al. proposed an interval-parameter fuzzy two-stage stochastic programming (IFTSP) model for effectively allocating water resources under uncertainty in order to balance the benefits of municipality, industry, and agriculture [26]. Another example is the two-stage mixed-integer fuzzy programming method with an interval-valued membership function proposed by Wang et al. for resolving the flood diversion planning problem under uncertainties [27]. Miao et al. proposed an interval-fuzzy de novo programming method for allocating water resources under uncertainties [28], while Khosrojerdi et al. suggested a two-stage interval-parameter stochastic fuzzy programming method to optimally allocate water resources to different users under uncertainties [29]. These examples show that the FMP method can effectively address fuzzy uncertainties in resource management systems [30,31,32]. Therefore, the FMP method was selected to handle the fuzzy uncertainty problems in the water resources system of MNNR.

In this context, in order to supplement the previous research and give consideration to interval parameter, random variable, and fuzzy variables in the system, an interval-parameter fuzzy two-stage stochastic programming (IPFTSP) model was constructed in the present study by incorporating ILP, TSP, and FMP. This IPFTSP model was subsequently applied for optimizing and adjusting the ecological water supplement scheme in MNNR under three scenarios, which shortened the scope of decision-making and reduced the decision-making risk. Another objective of the IPFTSP model was to maximize the restored suitable habitat area for the Siberian crane, oriental stork, and red-crowned crane in the MNNR. Meanwhile, the ecological and environmental benefits generated as a result of wetland restoration were taken into account and regarded as important constraints of the model. The results presented herein provide suggestions to managers for recovering wetland areas, protecting rare migratory water birds and their habitats, and formulating reasonable scientific optimization schemes for ecological water supplement.

## 2. Materials and Methods

### 2.1. Objective Function and Constraints of Interval-Parameter Fuzzy Two-Stage Stochastic Programming Model

Because of the complexity of the lake topography, the longitudinal profile of the lakes in MNNR was assumed to be a regular parabola to simplify the water level–area–volume relation curve. Moreover, the calculated function of the relationship coefficient between the suitable habitat area and the water volume was complex, but monotonically decreasing. In addition, the amount of water replenishment was limited, so the minimum value of the relationship coefficient was chosen to simplify the calculation. On the basis of the mentioned assumptions, fuzzy mathematical programming was added to the base of the IPTSP model, and the area of ecological function regions and the total planning area of lakes were set as fuzzy variables in order to obtain optimal solutions under further relaxed conditions. The fuzzy variables set in this study conform to the semi-trapezoidal membership function, defined as follows:

(1) $$\mu_{G}(x)=\{\begin{array}{cc} 0, & x\leq b\\ \frac{x-b}{a-b}, & b\leq x\leq a\\ 1, & x\geq a \end{array}$$

(2) $$0\leq \mu_{G}(x)\leq 1$$

where x represents fuzzy variable; $\mu_{G}(x)$ is the fuzzy membership degree corresponding to the fuzzy parameter; b represents the lower bound of the fuzzy variable; a represents the upper bound of the fuzzy variable. 

The objective function of the IPFTSP model is presented below:

(3) $$M\mathrm{ax}\;\lambda^{\pm }$$

where $\lambda^{\pm }$ represents the fuzzy membership interval of the model.

Constraints:

(1) The constraint for the maximization of the recovered habitat area is presented below:
  (4) $$\sum_{i=1}^{18}C_{i}^{\pm }\cdot QT_{i}^{\pm }-\sum_{i=1}^{18}\sum_{h=1}^{3}P_{h}\cdot C_{i}^{\pm }\cdot QS_{ih}^{\pm }\geq f^{+}-(1-\lambda^{\pm })\cdot (f^{+}-f^{-})$$
  where *i* represents the lakes in MNNR; $C_{i}^{\pm }$ denotes the relationship coefficient between the suitable habitat area and the water volume of lake *i* (ha/10^4^ m^3^); $QT_{i}^{\pm }$ is an output parameter of model, denoting the optimal water supply target for lake *i* (10^4^ m^3^); *h* represents the flood flow scenario;$P_{h}$ represents the scenario probability for scenario *h*, and $QS_{ih}^{\pm }$ represents the water shortage in the case of lake *i* under scenario *h* (10^4^ m^3^). $f^{+}$ and $f^{-}$ represent the upper and lower bound values of the recovered habitat area obtained from the IPTSP model in a previous study [10] (10^3^ ha).
(2) The constraint for minimum water demand is as follows:
  (5) $$QT_{i}^{\pm }-QS_{ih}^{\pm }\geq \sum_{j=1}^{4}QR_{ij}^{\pm }\cdot [A_{ij\min}^{+}-(1-\lambda^{\pm })\cdot (A_{ij\min}^{+}-A_{ij\min}^{-})],\forall i,h$$
  where *j* denotes the ecological function region, and j=1, 2, 3, 4 represent the fish pond, crab pond, reed, and marsh wetland, respectively; $A_{ij\min}^{\pm }$ is a fuzzy variable representing the lower boundary of the function region j of lake i (10^3^ ha); and $QR_{ij}^{\pm }$ represents the water demand per unit area for the function region *j* of lake i (10^4^ m^3^/10^3^ ha).
(3) The constraints for water supply capacity are as follows [10]:
  (6) $$QT_{i}^{\pm }-QS_{ih}^{\pm }\leq QID_{ih}^{\pm }+QND_{ih}^{\pm }+QFD_{ih}^{\pm }-QL_{i}^{\pm },\forall i,h$$
  (7) $$\sum_{i=1}^{9}QFD_{ih}^{\pm }\leq QTF_{nh}^{\pm },n=1,\forall h$$
  (8) $$\sum_{i=10}^{12}QFD_{ih}^{\pm }\leq QTF_{nh}^{\pm },n=2,\forall h$$
  (9) $$\sum_{i=13}^{16}QFD_{ih}^{\pm }\leq QTF_{nh}^{\pm },n=3,\forall h$$
  (10) $$\sum_{i=17}^{18}QFD_{ih}^{\pm }\leq QTF_{nh}^{\pm },n=4,\forall h$$
  (11) $$QID_{ih}^{\pm }\leq QI_{i}^{\pm },\forall i,h$$
  (12) $$QND_{ih}^{\pm }\leq QN_{i}^{\pm },\forall i,h$$
  where $QID_{ih}^{\pm }$, $QND_{ih}^{\pm }$ and $QFD_{ih}^{\pm }$ are output parameters of model, representing the local water supply, normal water supply, and the flood resource supply of lake *i* under scenario *h*, respectively (10^4^ m^3^); $QL_{i}^{\pm }$ denotes loss of water supply (10^4^ m^3^); *n* represents the water intakes in MNNR; $QTF_{nh}^{\pm }$ represents the available flood resources of water intake *i* under scenario *h* (10^4^ m^3^); $QI_{i}^{\pm }$ and $QN_{i}^{\pm }$ represent the local water supply and the normal water supply (10^4^ m^3^) [10].
(4) The constraints for water supplement sequence in different ecological function regions are as follows:
  (13) $$\sum_{j=3}^{4}FA_{ijh}^{\pm }\cdot QR_{ij}^{\pm }=\left\{ \begin{array}{l} QID_{ih}^{\pm }+QND_{ih}^{\pm }+QFD_{ih}^{\pm }-QL_{i}^{\pm }-\sum_{j=1}^{2}FA_{ijh}^{\pm }\cdot QR_{ij}^{\pm },\\ if\;\sum_{j=1}^{2}FA_{ijh}^{\pm }\geq \sum_{j=1}^{2}A_{ij\min}^{+}-(1-\lambda^{\pm })\cdot (A_{ij\min}^{+}-A_{ij\min}^{-})\\ 0\;,\;if\;\sum_{j=1}^{2}FA_{ijh}^{\pm }\leq \sum_{j=1}^{2}A_{ij\min}^{+}-(1-\lambda^{\pm })\cdot (A_{ij\min}^{+}-A_{ij\min}^{-})\; \end{array}\right.,\forall i,h$$
  (14) $$FA_{i4h}^{\pm }\cdot QR_{i4}^{\pm }=\left\{ \begin{array}{l} \sum_{j=3}^{4}FA_{ijh}^{\pm }\cdot QR_{ij}^{\pm }-FA_{i3h}^{\pm }\cdot QR_{i3}^{\pm },\\ if\;FA_{i3h}^{\pm }\geq A_{i3\min}^{+}-(1-\lambda^{\pm })\cdot (A_{i3\min}^{+}-A_{i3\min}^{-})\\ 0\;,\;if\;FA_{i3h}^{\pm }\leq A_{i3\min}^{+}-(1-\lambda^{\pm })\cdot (A_{i3\min}^{+}-A_{i3\min}^{-})\; \end{array}\right.,\forall i,h$$
  where $FA_{ijh}^{\pm }$ denotes the area of the ecological function region *j* of lake *i* (10^3^ ha). The constraints reflect that, in the project recommendation scheme, the fish and crab ponds are prioritized over the reed and marsh wetlands, while reed wetlands are prioritized over the marsh wetlands.
(5) The constraints for the area of ecological function regions are presented below:
  (15) $$\sum_{j=1}^{4}FA_{ijh}^{\pm }\leq TFA_{i}^{-}+(1-\lambda^{\pm })\cdot (TFA_{i}^{+}-TFA_{i}^{-}),\forall i,h$$
  (16) $$FA_{ijh}^{\pm }\leq A_{ij\max}^{-}+(1-\lambda^{\pm })\cdot (A_{ij\max}^{+}-A_{ij\max}^{-}),\forall i,j,h$$
  (17) $$\sum_{j=1}^{4}FA_{ijh}^{\pm }\geq C_{i}^{\pm }\cdot (QT_{i}^{\pm }-QS_{ih}^{\pm }),\forall i,h$$
  (18) $$\sum_{j=1}^{4}FA_{ijh}^{\pm }\cdot QR_{ij}^{\pm }=QT_{i}^{\pm }-QS_{ih}^{\pm },\forall i,h$$
  where $TFA_{i}^{\pm }$ is a fuzzy variable representing the total planning area of lake *i* (10^3^ ha), while $A_{ij\max}^{\pm }$ is the fuzzy variable representing the upper boundary of the ecological function region *j* of lake *i* (10^3^ ha).
(6) The constraint for the ecological benefits is presented below [10]:
  (19) $$\sum_{i=1}^{18}\sum_{j=1}^{4}FA_{ijh}^{\pm }\cdot Y_{ij}^{\pm }\cdot ESV_{jk}^{\pm }\geq TEB_{k}^{\pm },\forall k,h$$
  where *k* represents ecological service functions of MNNR; $Y_{ij}^{\pm }$ is a 0–1 variable; $ESV_{jk}^{\pm }$ represents the benefit per unit area of the ecological service function *k* of ecological function region *j* (10^6^ CNY/10^3^ ha); $TEB_{k}^{\pm }$ represents the ecological benefits of ecological service function *k* generated by the project recommendation scheme (10^6^ CNY) [10].
(7) The constraint for the net carbon sink is as follows [10]:
  (20) $$\sum_{j=1}^{4}FA_{ijh}^{\pm }\cdot Y_{ij}^{\pm }\cdot NCSA_{ij}^{\pm }\geq TCS_{i}^{\pm },\forall i,h$$
  where $NCSA_{ij}^{\pm }$ denotes the net carbon sink capacity of ecological function region *j* (t/10^3^ ha); $TCS_{i}^{\pm }$ denotes the net carbon sink of lake *i* generated by the project recommendation scheme (t) [10].

### 2.2. Parameters and Solution of Interval-Parameter Fuzzy Two-Stage Stochastic Programming (IPFTSP) Model 

In the present study, the parameters used to determine the solution of the IPFTSP model were consistent with those used to determine the solution of the IPTSP model in a previous study [10]. Data on the parameters used in the present study were obtained mainly from the literature [33], whose data were project data obtained from the West Water Supply Project of Jilin Province. 

In order to determine the solution of the IPFTSP model, the objective function of the IPTSP model ($f^{+}$ and $f^{-}$) was obtained and subsequently substituted into the IPFTSP model as a constraint condition, which provided the solutions. With the adoption of an interactive algorithm for a solution, the IPFTSP model was transformed into two groups of deterministic sub-models, which corresponded to the upper and lower bounds of the objective function [34]. The upper bound and lower bound sub-models correspond to the optimal solution under optimal conditions and the worst solution under the worst conditions, respectively. In the present study, the Lingo 11.0 software was employed to determine the solutions of the two sub-models of the IPFTSP model, and the obtained solutions of the model were: *QT_i_*, $\lambda_{opt}^{\pm }=[\lambda_{opt}^{-},\lambda_{opt}^{+}]$, $QS_{ihopt}^{\pm }=[QS_{ihopt}^{-},QS_{ihopt}^{+}]$. In addition, the optimal water supplement schemes were: $Q_{ihopt}^{\pm }=QT_{i}-QS_{ih}^{\pm },\forall i,h$. On the basis of these model solutions, the recovered suitable habitat area corresponding to this value was calculated and determined to be $HA_{opt}^{\pm }=[HA_{opt}^{-},HA_{opt}^{+}]$.

## 3. Case Study

**Figure 1:**
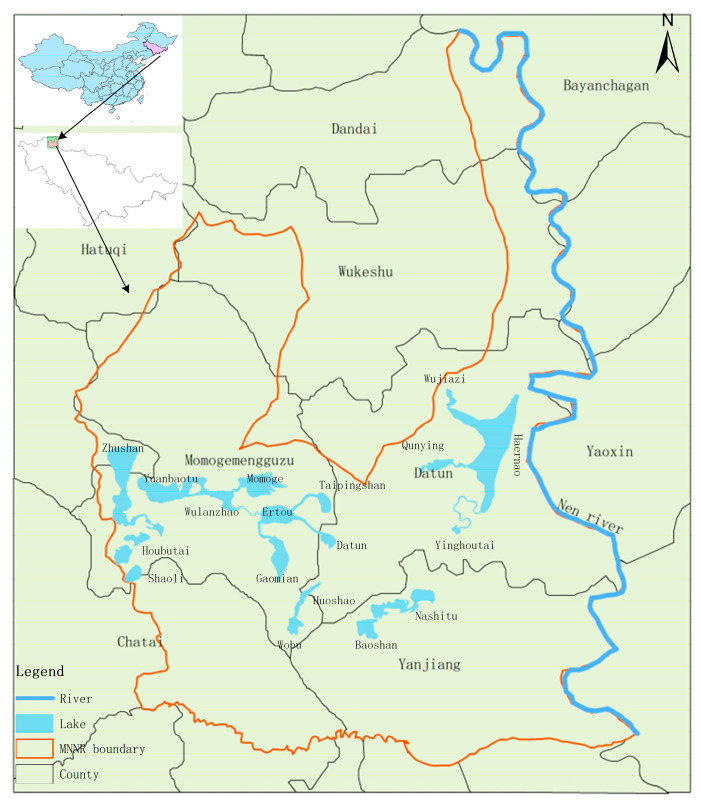
Map of Momoge National Nature Reserve (MNNR).

**Figure 2:**
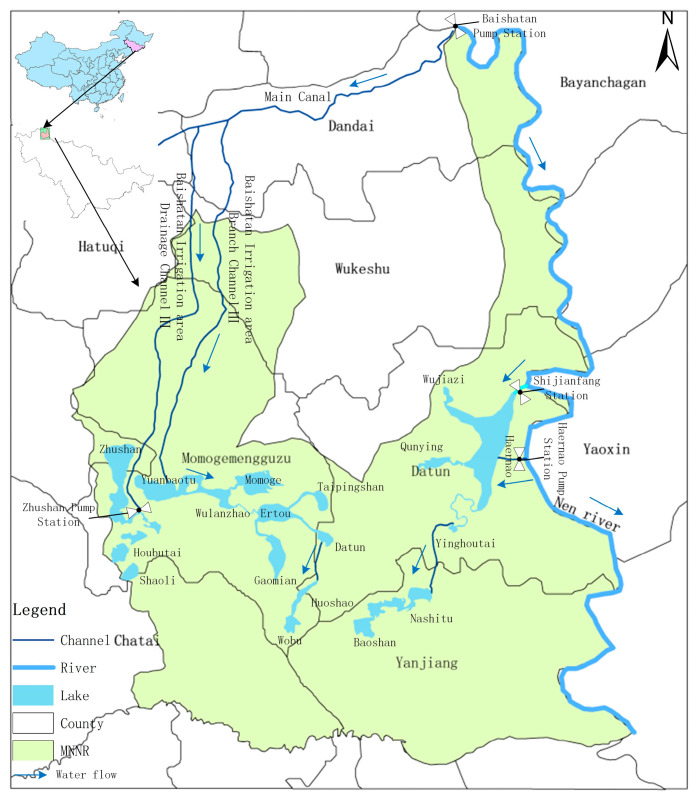
Schematic diagram of water supplement route in Momoge National Nature Reserve (MNNR).

Momoge National Nature Reserve (MNNR) is the largest wetland reserve of Jilin Province, with a total area of 14.4 × 10^4^ ha, which is rich in animal and plant resources. Its main protection objects are inland wetlands, aquatic ecosystem, and rare water birds including the Siberian crane, oriental stork, red-crowned crane, and great bustard. In MNNR, the average annual rainfall is less than 400 mm, but the evapotranspiration is up to 900–1000 mm. Moreover, the average annual temperature is gradually rising, resulting in the natural shortage of water resources in MNNR. In addition, due to the destruction caused by human factors such as engineering construction, the wetland in MNNR has been seriously degraded and the area of the wetland has been greatly reduced. In comparison to the 1970s, the wetland area of MNNR in 2012 had reduced by >50% [9]. As a result, the area of suitable habitat for rare migratory water birds has also been greatly reduced, leading to a sharp decrease in the number of rare water birds inhabiting MNNR. In order to recover and protect the wetland in MNNR, the West Water Supply Project of Jilin Province included in the recommended scheme prioritizes the water supplement in MNNR through the “Yinnenrubai” water supplement project. In this study, 18 lakes in the MNNR were selected as the study area, shown in Figure 1. The project recommendation scheme plans to supply a normal water supplement of 7633 × 10^4^ m^3^, a local water supplement of 7592 × 10^4^ m^3^, and flood resources of 4518 × 10^4^ m^3^ to the MNNR, and to restore 34.23 × 10^3^ ha of the wetland area. The water supplement node and the water supplement route in the MNNR are shown in Figure 2.

## 4. Results and Discussion

### 4.1. Analysis of the Optimization of Ecological Water Supplement Scheme 

#### 4.1.1. Analysis of the Optimization of Ecological Water Supplement Scheme Using Interval-Parameter Fuzzy Two-Stage Stochastic Programming (IPFTSP) Model

By solving the IPFTSP model, the fuzzy membership interval was obtained as λ = (0.01, 0.83). In addition, the optimal targets of water supply, the local and normal water supply, and the flood resource supply, under the three scenarios, were obtained and are presented in Table 1. 

As shown in Table 1, the total water supply target of the four water intakes in MNNR of the IPFTSP model was 24,924.81 × 10^4^ m^3^ (the first-stage decision), with a decrease of 5.56% compared to that of IPTSP model, which is conducive to reducing water use [10]. The actual water supply is the sum of the local and normal water supply, and the flood resource supply. According to Table 1, the total actual optimal water supplements under the scenarios of low, medium, and high flood flow were calculated to be (18,833.44, 22,250.48) × 10^4^ m^3^, (20,201.00, 23,696.41) × 10^4^ m^3^, and (21,910.44, 24,161.79) × 10^4^ m^3^, respectively. Furthermore, the water shortage (the second-stage decision) is calculated as the difference between the actual water supply and the optimal target of water supply. The water shortages under low, medium, and high flood scenarios were determined to be (2674.33, 6091.38) × 10^4^ m^3^, (1228.41, 4723.82) × 10^4^ m^3^, and (763.03, 3014.37) × 10^4^ m^3^, respectively, demonstrating that the optimal water supply targets under the three scenarios were not fulfilled. When the water shortages under different scenarios were compared, it could be observed that, with the increase in flood flow, the water shortages at different water intakes decreased gradually, while the total amount of optimal water supplement continuously increased. This observation demonstrated that the developed IPFTSP model was capable of effectively optimizing and adjusting the ecological water supplement scheme recommended by the project, and could completely utilize the limited water resources and avoid wastage of water resources under various scenarios. 

The optimization of the ecological water supplement scheme gave priority to the supplement of flood resources and improved the utilization of flood resources. In the total actual optimal water supplement, the proportions of flood resource supply under the three different scenarios were (27.49%, 29.05%), (33.85%, 36.87%), and (39.01%, 43.39%), respectively. In addition, the proportion of flood resource supply increased with the increase in flood flow, indicating that complete utilization of the flood resources under various scenarios could meet the water demand of MNNR and alleviate the water shortage to a certain extent [35]. Furthermore, the ecological water replenishment scheme after optimization could make full use of flood resources and improve the utilization rate of transit flood resources in MNNR, which is supported by conclusions reported by Liu [9] and Qiu et al. [11]. Further, under the three different scenarios, the interval ranges of the actual optimal water supply were observed to be 18.14%, 17.30%, and 10.28% respectively, which is similar to the research result reported by Cai et al., that the interval range of the model result was 9.23% [36]. This observation indicated that the model optimization results obtained through the ILP method could offer a certain decision-making space to the managers of nature reserves in adjusting the decision scheme.

**Figure 3:**
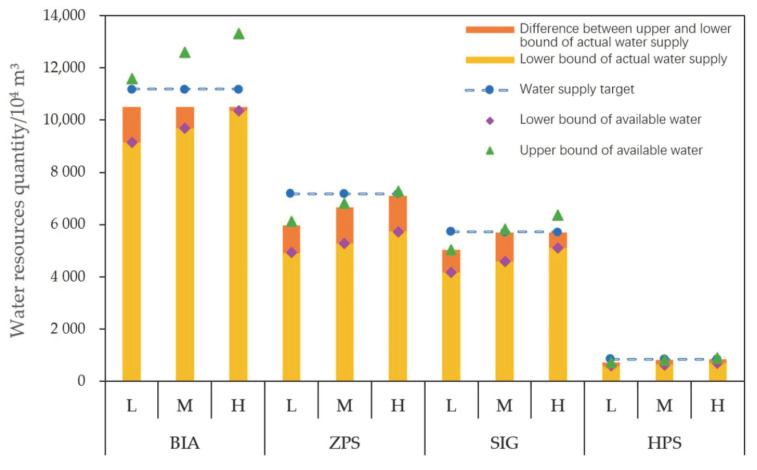
Comparison of the actual optimal water supply and the available water of the four water intakes under different flood flow scenarios. Note: BIA = third branch channel of the Baishatan irrigation region; ZPS = Zhushan pumping station; SIG = Shijiangfang intake gate; and HPS = Haernao pumping station. L, M, and H denote low, middle, and high flood flow scenarios, respectively.

Figure 3 illustrates the size relationship between the actual water supply and the total available water of the four water intakes in MNNR under the three different flow scenarios. As illustrated in Figure 3, under the scenarios of low, medium, and high flood flow, the lower bound values of the actual optimal water supply of the BIA were all identical to the lower bound values of the total available water, while the upper bound values of the total available water were all greater than the upper bounds of the actual optimal water supply, with the differences of 1063.39 × 10^4^ m^3^, 2097.62 × 10^4^ m^3^, and 2787.10 × 10^4^ m^3^, respectively. In addition, the upper bound values of the total available water were greater than the optimal target of water supply by 386.85 × 10^4^ m^3^, 1421.08 × 10^4^ m^3^, and 2110.56 × 10^4^ m^3^ under the three different scenarios, respectively, although water shortages nonetheless remained. This observation is mainly because the scope of planning in the case of the lakes in MNNR is limited and cannot be expanded indefinitely, and consequently, the acceptable amount of the ecological water supplement is also limited. The optimal water supply targets of certain lakes corresponding to the BIA were greater than the acceptable ecological water supply, leading to water shortage even when the water supply was adequate. Therefore, the optimal water supply targets of these lakes require adjustment. At the same time, the upper bound value of the total available water was higher than the optimal water supply target, which also indicated that the system had a strong capability to meet the actual optimal water supply, causing the risk of system failure to be relatively low. The same law is also reflected in the ZPS. Under the three different flow scenarios, the total available water was equal to the actual water supply in the lower bound sub-model, while the upper bound sub-model had a small surplus water resources. In the case of the SIG, the optimal water supply target could only be met under the medium and high flood flow scenarios in the upper bound sub-model, with a water surplus of 141.00 × 10^4^ m^3^ and 669.31 × 10^4^ m^3^, respectively. The optimal water supply target of the HPS could be met only under the scenario of high flood flow in the upper bound sub-model, with a water surplus of 43.49 × 10^4^ m^3^. Overall, under the three flood flow scenarios, the ecological water supplement in the lower bound sub-model was inadequate, and was required to be increased, while there was a water surplus of 1201.75 × 10^4^, 2376.97 × 10^4^, and 3659.03 × 10^4^ m^3^, respectively, in the upper bound sub-model. It is recommended to direct the surplus water toward other regions with water demand in order to avoid wastage of water resources.

#### 4.1.2. Comparison of the Optimization of Ecological Water Supplement Scheme between IPFTSP and IPTSP Models

In comparison to the IPTSP model [10], the FMP method reduced the interval ranges of actual optimal water supply in MNNR of the IPFTSP model under different flood flow scenarios, resulting in a reduction of the decision-making scope. As shown in Table 2, the lower bound values of actual optimal water supply in the IPFTSP model are equal to those in the IPTSP model, while the upper bound values in the IPFTSP model are significantly smaller than those in the IPTSP model. The interval ranges of actual optimal water supply under the three different flood flow scenarios in the IPFTSP model were reduced by 26.02%, 31.74%, and 49.62% compared to those in the IPTSP model, and the reduced part belonged entirely to the upper bound values of actual optimal water supply. This was conducive to reducing the system failure risk of the required water supply not being met. After completing the analysis, it was deduced that under the influence of the membership function, the upper bound constraint value of the total planning area of the lakes in the sub-model of the IPFTSP model had reduced to a certain extent compared to that in the IPTSP model, which resulted in the decrease in the upper bound value of acceptable ecological water supplement. While the total planning area constraint of the lakes in the lower-bound sub-model of the IPFTSP model was also reduced, the lower bound value of actual optimal water supply was not reduced as there was an insufficient ecological water supply. The decrease in the actual optimal water supply interval in the IPFTSP model would result in an obvious reduction in the interval range of the recovered area of an ecological function region and the suitable habitat for rare migratory water birds, which is conducive to reducing the decision-making risk as well as the decision-making scope and assisting the decision-makers in formulating a reasonable ecological water supply scheme.

### 4.2. Analysis of Habitat Area and Carrying Capacity for Rare Migratory Water Birds Recovered Using Ecological Water Supplement Scheme Optimization under Different Flood Flow Scenarios

#### 4.2.1. Analysis of Habitat Area Recovered Using IPFTSP Model

Using ecological water supplement optimization schemes under different flood flow scenarios and the relationship coefficients $C_{i}^{\pm }$, the recovered habitat areas of the eighteen lakes in MNNR were determined and are presented in Table 3. Here, for the sake of convenience, the lake groups corresponding to the water intakes BIA, ZPS, SIG, and HPS are designated as regions r = 1, 2, 3, and 4, respectively.

As stated earlier, fuzzy membership reflects the possibility that the model scheme fulfills the fuzzy objectives and constraints [26], which also implies the possibility of obtaining the model target value. According to Table 3, the target value of the suitable habitat area for rare migratory water birds recovered through the IPFTSP model was (14.60, 18.47) × 10^3^ ha, with a possibility level of (0.01, 0.83), which reflected the level of system failure risk. Under the three flood flow scenarios, the recovered habitat area values obtained through the upper bound model scheme were 17.88 × 10^3^ ha, 18.96 × 10^3^ ha, and 19.43 × 10^3^ ha, while the target value was 18.47 × 10^3^ ha, with a possibility level of 0.83 that indicated a high level of system failure risk. The recovered habitat area values obtained through the lower bound model scheme were 14.06 × 10^3^ ha, 14.92 × 10^3^ ha, and 15.83 × 10^3^ ha, while the target value was 14.60 × 10^3^ ha, with a possibility level of 0.01, which indicated a significantly reduced risk of system failure. In other words, if a decision-maker selects the target valve of 18.47 × 10^3^ ha at a probability level of 0.83, it presents a high risk in the corresponding decision scheme; in comparison, if the decision-maker selects the target valve of 14.60 × 10^3^ ha at a probability level of 0.01, it sharply reduces the risk in the decision scheme. 

The model target value and fuzzy membership would vary within their intervals as the parameters in the IPFTSP model are adjusted within the interval [29]. Clarifying the changing relationship between the model target value and fuzzy membership assists the decision-makers to make the preferred decision. In order to determine the relationship between the size of recovered habitat area for rare migratory water birds and the corresponding risk level, the following situations were set for the analysis: (1) the optimization scheme presented in Table 3 was designated as situation 1; (2) situation 2 involved taking the available flood resources as the upper bound value in the lower bound sub-model; and (3) situation 3 involved taking the available flood resources as the lower bound value in the upper bound sub-model [the available flood resources are presented in Table 2 of reference [10]. According to the solution, the target value determined in situation 2 was 15.28 × 10^3^ ha with a possibility level of 0.16, while the target value determined for situation 3 was 18.02 × 10^3^ ha with a possibility level of 0.74. In this case, when the lower bound value of the target value in situation 1 was compared with that of the target value in situation 2, it was observed that the target value in situation 2 was larger and the corresponding possibility level had also increased. This could be attributed to the increase in the flood resources, because of which the scheme of situation 2 exhibited a higher capability of meeting the target value and constraints, resulting in a greater target value and a higher possibility of satisfying the target value and constraints. However, the increase in flood resource utilization would also present a higher level of system failure risk, i.e., the demand for flood resource utilization cannot be met. The same law might be observed while comparing the target value in situation 3 with the upper bound value of the target value in situation 1. In situation 3, the utilization of flood resources was reduced, and a part of the target value was sacrificed in order to reduce the level of system failure risk. The fuzzy membership assists the decision-makers to tradeoff the relationship among the target value, the risk level, and the possibility level during the formulation of the preferred water supplement scheme.

The suitable habitat area recovered through the ecological water supplement schemes optimized using the IPFTSP model increased, compared to the ecological water supplement recommended by the project. As shown in Table 3, the target value of the recovered habitat area, as a whole, in the IPFTSP model, exhibited a significant increase of (9.23%, 38.25%) compared to the value of 13.36 × 10^3^ ha in the project recommendation scheme. Under different flood flow scenarios, the habitat area recovered through optimal water supplement schemes exhibited improvement in comparison to that in the recommended scheme, with the increases of (5.22%, 33.78%), (11.62%, 41.88%), and (18.44%, 45.39%) at the scenario probabilities of 50%, 38%, and 12%, respectively. In addition, the recovered habitat areas under the scenarios of medium and high flood flow were significantly increased by (6.04%, 6.12%) and (8.67%, 12.59%), respectively, compared to that under the low flood flow scenario. After optimizing allocation through the IPFTSP model, the increased water resources observed with the increase in flood flow could be utilized effectively, which could alleviate water shortage as well as increase the recovered habitat area for rare migratory water birds under the different scenarios, with significant optimization effect. Locally, the size relationship of the recovered habitat area in different regions under the different flood flow scenarios was as follows: r = 1 > r = 2 > r = 3 > r = 4. In addition, under the three flood flow scenarios, the upper bound values of the habitat area recovered through optimal water supplement schemes exhibited improvement in all the regions in comparison to the corresponding values in the recommended scheme, while the lower bound values of the recovered habitat area in regions 2 and 4 exhibited a relative decrease. This was mainly because of inadequate water supply in regions 2 and 4 in the lower bound sub-model, and it is, therefore, recommended to divert water from other places to these regions. 

#### 4.2.2. Analysis of Carrying Capacity Recovered Using IPFTSP Model

The Siberian crane, oriental stork, and the red-crowned crane migrate to MNNR in the autumn season (from early September to early October), where they rest for 1 to 2 months [37]. Thus, the optimum slot for ecological water supplement in MNNR is from July to September, with the aim of accomplishing the objective before the arrival of these rare migratory water birds. According to the research results reported by Xiao, the distribution density of Siberian crane in the suitable habitat of MNNR is estimated to be 0.63–0.89 ha/bird, with the average value of 0.76 ha/bird [38]. Since the study species were similar in body size, their distribution densities could be regarded as the same. On the basis of this, for calculation in the present study, 0.76 ha/bird was used as the distribution density of the study species in suitable habitats. 

**Figure 4:**
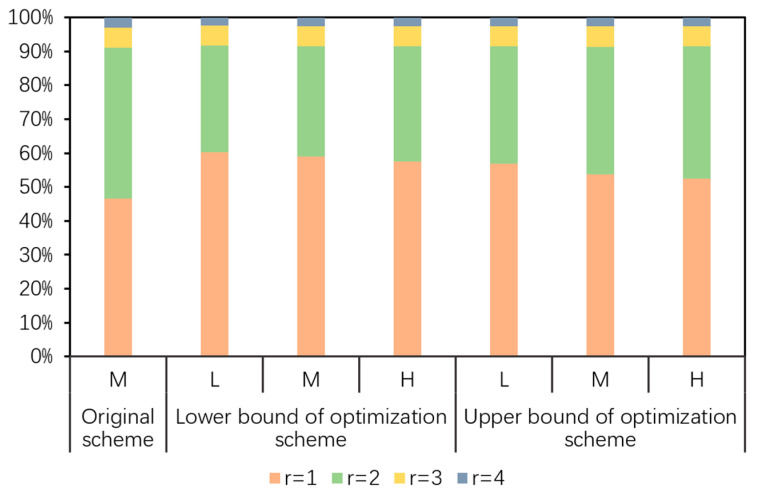
Shares of the carrying capacities of different regions for rare migratory water birds under different scenarios. L, M, and H denote low, middle, and high flood flow scenarios, respectively.

Using the distribution density of the study species and the recovered habitat area in each lake, it was possible to determine the carrying capacity of MNNR for the study species. The carrying capacity for the study species recovered through the optimization schemes under the different flood flow scenarios was (18.50, 23.52) × 10^3^, (19.63, 24.95) × 10^3^, and (20.83, 25.56) × 10^3^ birds, with the increase of (5.22%, 33.78%), (11.62%, 41.88%), and (18.44%, 45.39%), respectively, compared to the recommended scheme. These increased proportions clearly indicated that the ecological water supplement scheme optimization was capable of improving the carrying capacity for rare migratory water birds in MNNR, which would facilitate the recovery and expansion of the populations of rare migratory water birds; this has important implications in the safety of endangered and rare species of migratory water birds such as the Siberian crane [39]. As shown in Figure 4, under different flood flow scenarios, the carrying capacity for rare migratory water birds recovered through the ecological water supplement scheme before and after the optimization was the largest for regions 1 and 2, together accounting for greater than 90%, which indicated that in regions 1 and 2, the recovered suitable habitat, as well as the number of members of the study species that could be accommodated, was the largest, resulting in a higher probability of inhabitation of the study species in these regions.

#### 4.2.3. Comparison of Recovered Habitat Area and Carrying Capacity between IPFTSP and IPTSP Models

**Figure 5:**
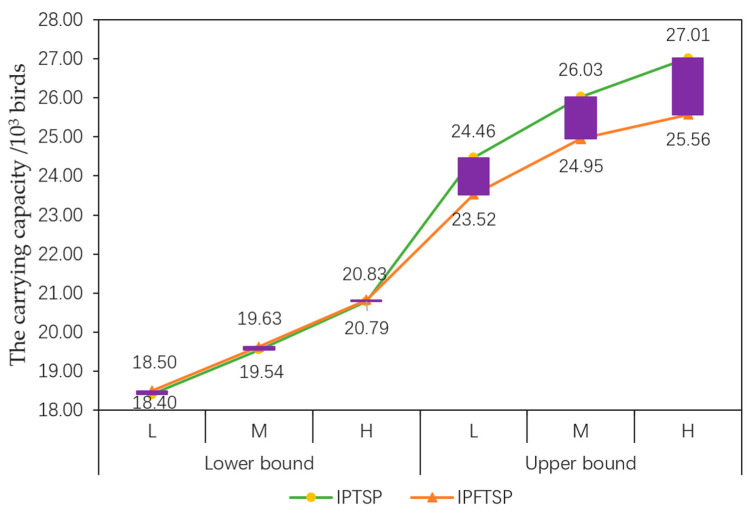
Comparison of the recovered carrying capacity for rare migratory water birds between the IPFTSP and IPTSP models under different flood flow scenarios. L, M, and H denote low, middle, and high flood flow scenarios, respectively.

The recovered habitat area and carrying capacity for rare migratory water birds are consistent parameters. Therefore, the influence of fuzzy mathematical programming on the optimization schemes could be analyzed through a comparison between the IPFTSP and IPTSP models in terms of the recovered habitat area and the carrying capacity for rare migratory water birds. Table 4 and Figure 5 present the comparisons of the recovered habitat area and the carrying capacity for rare migratory water birds in MNNR between the IPFTSP and IPTSP models, respectively, under different scenarios. Figure 5 reveals that the recovered carrying capacity for rare migratory water birds in the IPTSP model was (18.40, 24.46) × 10^3^, (19.54, 26.03) × 10^3^, and (20.79, 27.01) × 10^3^ birds, with increases of (4.66%, 39.14%), (11.15%, 48.04%), and (18.24%, 53.63%), respectively, compared to the project recommendation scheme. The comparison of the recovered habitat area and the carrying capacity for rare migratory water birds between the IPTSP and IPFTSP models revealed that under different flood flow scenarios, the upper bound values of the recovered habitat area and the carrying capacity in the IPFTSP model were smaller than those in the IPTSP model, while the lower bound values were all greater than the corresponding values in the IPTSP model. This was because the IPFTSP model adds a fuzzy programming method to the base of the IPTSP model, which is capable of completely considering the fuzzy uncertainty of the area of the ecological function region and total planning area of the lakes, and then optimize the constraint conditions through membership relationship, thereby increasing the lower bound values and reducing the upper bound values of the recovered habitat area and the carrying capacity obtained earlier in the IPTSP model. 

The interval width of the target value in the IPFTSP model was smaller than that in the IPTSP model [40]. In the case of the three different flood flow scenarios, the interval ranges of the recovered suitable habitat area and the carrying capacity obtained in the IPFTSP model were reduced by 17.15%, 17.98%, and 23.86%, while the interval range of the target value was reduced by 18.28%, compared to the IPTSP model. In the report by Maqsood et al. [26], the reduced scale of the interval range of the target value in the IFTSP model was 24.10%. Therefore, the narrowing interval range of the target value obtained in the present study is similar to that reported by Maqsood et al. [26]. In the IPTSP model, the constraints of the lower bound sub-model are strict, and therefore, there is a risk of wastage of the limited water resources and reduction of the target value due to being too conservative. On the other hand, the constraints of the upper bound sub-model are lenient, and therefore, there is a risk of system failure due to excessive demand for water that the system is incapable of meeting. The IPFTSP model with a fuzzy programming method is capable of reducing the upper bound values of the model. This can reduce the corresponding system failure risk and improve the lower bound values of the model to reduce the risk of wastage of the limited water resources. Therefore, compared to the IPTSP model [10], the FMP method reduced the interval ranges of the IPFTSP model result to narrow the scope of decision-making and avoid the two extreme cases with the highest risk of water wastage and system failure, ultimately reducing the decision-making risk. In addition, the fuzzy membership $\lambda^{\pm }$ of the IPFTSP model reflects the relationship between the target value and the risk level, which could provide decision support for the formulation of ecological water supplement schemes with a reasonable target value and risk level [26].

### 4.3. Analysis of the Area of Lake and Wetland Recovered Using Ecological Water Supplement Scheme Optimization under Different Scenarios

#### 4.3.1. Analysis of the Area of Lake and Wetland Recovered Using IPFTSP Model

Ecological water supplement is an important and efficient approach for the restoration of wetlands [8]. The ecological water supplement scheme optimized using the IPFTSP model improved the recovered area of the lakes and wetlands in MNNR, compared to the recommended scheme. According to Table 5, the recovered water surface areas (fish pond and crab pond) in the optimal schemes under the three flood flow scenarios were (8.13, 11.75) × 10^3^ ha, (10.49, 12.52) × 10^3^ ha, and (12.11, 12.52) × 10^3^ ha, with the changes of (−17.11%, 19.74%), (6.89%, 27.56%), and (23.44%, 27.56%), respectively, compared to the recommended scheme. The ecological water supplement in MNNR would be accomplished before the arrival of the rare migratory water birds. Therefore, the increase of water surface area was beneficial to increase the suitable habitat area of rare water birds. In addition, the water supplement originates from the Nenjiang River, and a large amount of fish, crabs and other foods would be carried in the water supplement, which provided abundant food resources for rare water birds. The larger area of lakes and wetlands as well as abundant food resources were conducive to the survival of rare water birds, which would attract more rare water birds. Among these increases, the lower bound value of the recovered water surface area exhibited a great increase, while the upper bound value either increased slightly or remained unchanged. The restoration of water surface area has a close association with the ecological water supplement. Since the ecological water supplement in the lower bound sub-model under different scenarios is inadequate, the recovered water surface area increases significantly with the increase in water supplement. On the contrary, the water supplement in the upper bound sub-model under the three flow scenarios is relatively sufficient with just small differences, and the area of the fish pond and crab pond is limited, which results in either a small increase or no increase at all in the upper bound value of the recovered water surface area. 

The restoration of larger wetland areas (reed and marsh wetlands) could provide more nesting sites and sufficient food resources such as bulbs of *Scirpus planiculmis* for rare water birds. The recovered wetland areas under the three flood flow scenarios were (21.17, 26.48) × 10^3^ ha, (20.82, 28.30) × 10^3^ ha, and (21.46, 29.26) × 10^3^ ha, with the changes of (−13.29%, 8.42%), (−14.73%, 15.90%), and (−12.12%, 19.83%), respectively, compared to the recommended scheme. It was observed that the lower bound value of the recovered wetland area under the three flood flow scenarios was smaller than that of the recommended scheme. After analysis, the main reasons identified for this were as follows: (1) in the order of water supplement priority, the fish and crab ponds came prior to the wetlands, and therefore, under the condition of inadequate water supplement, the water demand of the water surface area needs to be satisfied first; (2) the ecosystem service value per unit area of fish and crab ponds is higher than that of the wetlands, while the ecological water demand per unit area of the ponds is lower than that of reed wetland, which implies that in the case of insufficient water supply, supplying more water to fish and crab ponds is conducive to enhancing the overall ecological service value of the system. The changes in the recovered wetland area under the scenarios of medium and high flow were (−1.66%, 6.89%) and (1.36%, 10.53%), respectively, compared to that under the low flood flow scenario, which implies that the lower bound value of the recovered wetland area under the medium flow scenario is lower than the corresponding value under the low flow scenario. This could be attributed to the fact that the water demand per unit area for reed wetland is greater than that for the crab pond and the marsh wetland, while its ecosystem service value per unit area is less than that of the crab pond. Therefore, based on increasing the area of marsh wetland in order to satisfy the constraint for net carbon sink, appropriate reduction in the reed wetland area in the case of inadequate water supplement is conducive not only to increasing the ecosystem services value but rather also to increasing the total restoration area.

#### 4.3.2. Comparison of the Recovered Area of Lake and Wetland between IPFTSP and IPTSP Models

In Table 6, we present the total area of ecological function region in MNNR recovered through IPFTSP and IPTSP models under different flood flow scenarios. As shown in Table 6, compared to the recovered area of 34.23 × 10^3^ ha in the recommended scheme, the lower bound values of the total area of the function region recovered through IPTSP and IPFTSP models were smaller (in both models), while the upper bound values were greater. This could be mainly attributed to the characteristics of the interval-parameter method used. The lower bound value is obtained under the worst conditions (e.g., the ecological water demand per unit area in the lower bound sub-model is greater than that in the project recommended scheme), and the water supply is inadequate, resulting in smaller lower bound values of the total recovered area of the function region in the IPFTSP and IPTSP models compared to those in the recommended scheme. In the IPFTSP model, the total recovered areas of 38.23 × 10^3^ ha, 40.82 × 10^3^ ha, and 41.78 × 10^3^ ha under different scenarios were obtained at a probability level of 0.83, which reflects a high degree of satisfaction for the managers although with a high level of system failure risk. On the other hand, the total recovered areas of 29.31 × 10^3^ ha, 31.31 × 10^3^ ha, and 33.57 × 10^3^ ha under different scenarios were obtained at a probability level of 0.01, which reflects a significantly reduced risk of system failure and an extremely low degree of satisfaction for the managers. 

**Figure 6:**
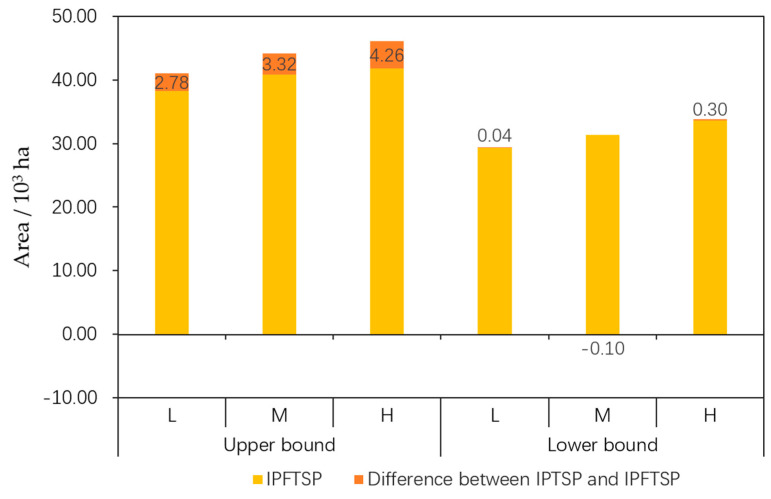
Comparison of the total recovered area of the ecological function regions between IPFTSP model and IPTSP model under different flood flow scenarios. L, M, and H denote low, middle, and high flood flow scenarios, respectively.

The FMP method reduced the interval ranges of the total area of ecological function regions of the IPFTSP model to narrow the decision-making scope, compared to the IPTSP model [10]. As observed in Figure 6, under different scenarios, the lower bound values of the gross recovered area in the IPFTSP model were almost equal to those in the IPTSP model, while the upper bound values were significantly smaller compared to those in the IPTSP model, with the differences of 2.78 × 10^3^ ha, 3.32 × 10^3^ ha, and 4.26 × 10^3^ ha, respectively. This led to a significant reduction in the interval width of the total recovered area in the IPFTSP model compared to that in the IPTSP model, and the reduction ratios under the different scenarios were 23.52%, 26.48%, and 32.54%, respectively (Table 6). The reduction in the interval width is influenced mainly by the fuzzy programming method used. The optimization of constraints with the membership function by the fuzzy programming method significantly reduced the upper bound value of the actual water supply in the IPFTSP model, resulting in the decrease in the upper bound value of the total recovered area in the IPFTSP model compared to that in the IPTSP model, although with the benefits of narrowing the scope of decision-making and reducing the level of risk of the actual total recovered area not reaching its upper bound value. The reason for the lower bound value of the total recovered area in the IPFTSP model being almost equal to that in the IPTSP model is that the lower bound values of the actual optimal water supply in the IPFTSP and IPTSP models are equal and in the state of shortage with just a small adjustable space. As a consequence, the lower bound value of the total recovered area varies slightly between the two models. The reduction of the decision-making scope would make decision selection easier.

### 4.4. Calculation of Ecological Service Value and Net Carbon Sink Generated by the Ecological Function Region Recovered through Multiple Water Supplement Schemes under Different Flood Flow Scenarios

In the IPFTSP model constructed in the present study, in addition to considering the recovery of the suitable habitat area of rare migratory birds, the ecosystem service value and net carbon sink, were included as part of the important constraints, in order to provide greater ecological and environmental benefits while obtaining maximum recovered suitable habitat area. Table 7 presents the ecological service value and the net carbon sink generated by the recovered ecological function region in the recommended scheme and through the optimization of water supplement schemes using the IPFTSP and IPTSP models under different flood flow scenarios. As visible in Table 7, in comparison to the project recommendation scheme, the variation ranges of the ecological service value generated in the IPFTSP model were (−14.24%, 35.18%), (−7.01%, 44.02%), and (0.29%, 47.10%) under different scenarios, while the changes in the net carbon sink generated in the IPFTSP model were (−12.00%, 36.54%), (−17.40%, 42.74%), and (−9.19%, 44.24%). Under the three flood flow scenarios of the IPFTSP model, the upper bound values of the ecological service value and the net carbon sink were determined at a probability level of 0.83, which reflects a high level of system failure risk, while the lower bound values were obtained at a probability level of 0.01, which reflects a sharply reduced risk of system failure. 

When the ecological service value and the net carbon sink generated were compared between the IPFTSP and IPTSP models, it was observed that under different flood flow scenarios, the lower bound values of the ecological service value and the net carbon sink generated in the IPFTSP model differed slightly from those in the IPTSP model, while the upper bound values were significantly smaller than those in the IPTSP model. The change of the upper bound values of ecological service value in the present study is similar to the research result of Cai et al., that the ecological service value obtained after adding the fuzzy mathematical programming decreased by 6.79% compared to the original plan [41]. As a consequence, the interval width of the ecological service value and the net carbon sink in the IPFTSP model were also reduced compared to that in the IPTSP model, with the reductions of 13.39%, 16.59%, and 21.86% in the ecological service value and the reductions of 16.41%, 23.14%, and 28.08% in the net carbon sink (Table 7). In comparison to the IPTSP model [10], the FMP method reduced the interval width of the ecological service value and the net carbon sink in the IPFTSP model, which lowered the risk of the actual recovered ecological service value and net carbon sink not reaching the upper bound values, and shortened the decision range to make decision selection easier.

## 5. Conclusions

In the present study, an interval-parameter fuzzy two-stage stochastic programming (IPFTSP) model was constructed through the addition of fuzzy mathematical programming to the base of interval-parameter two-stage stochastic programming (IPTSP) model, with the aim of effectively addressing the interval parameters, random variable, and the fuzzy variables in the system simultaneously, to narrow down the decision-making scope, ultimately reducing the decision-making risk. In addition, the constructed IPFTSP model was applied for the optimization and adjustment of the ecological water supplement scheme in Momoge National Nature Reserve, with the objective of obtaining maximum recovered habitat area for the Siberian crane, oriental stork, and red-crowned crane, and for generating better ecological service value and net carbon sink. 

This study demonstrated that the constructed IPFTSP model could reasonably utilize water resources under different flood flow scenarios, thereby resolving the issue of wastage of water resources in the case of sufficient availability of water resources. This was well reflected in the results when the recovered habitat area and the carrying capacity for rare migratory water birds were observed to increase with the increase in flood flow. In addition, the upper bound values of the recovered habitat area and the carrying capacity under different flood flow scenarios were obviously larger than the lower bound values, offering decision-making space to the decision-makers for adapting the ecological water supplement optimization scheme to the actual requirements. In contrast to the IPTSP model, the added FMP method reduced the overall decision-making range of the IPFTSP model by 18.28%, leading to a significant reduction in the upper bound values of the ecological service value, the net carbon sink, the recovered area of the water surface and the wetlands, the recovered habitat area, and the carrying capacity for rare migratory water birds in the IPFTSP model, which was achieved with a reduced system risk of not being able to reach the upper bound values in the model result in practice. In addition, the reduction in the decision–making scope in the IPFTSP model brings the model results closer to the actual situation and reduces the decision-making risk, which assists the managers in undertaking reasonable decisions conveniently. Moreover, the obtained fuzzy membership of the IPFTSP model reflected the relationship among the target value, the possibility level, and the risk level, which could provide decision support to the decision-makers for formulating the water resource management scheme with a reasonable target value and an appropriate risk level. The increase in the numbers of rare migratory water birds would be conducive to the study of their ecological habits and migration rules, which is another among the important significances of the present study. 

Despite the promising results, the present investigation has certain limitations. One limitation is that besides water depth and potential food resources, no other factors such as total wetland area, total water surface area, and human disturbances were taken into consideration while defining the suitable habitat area for rare migratory water birds [42]. This is because the data on the impact of these factors on suitable habitat areas is lacking. Furthermore, the food sources favored by rare water birds such as fish, crab and *Scirpus planiculmis* were not directly considered in this study. In addition, certain assumptions were made during the calculation of the relationship coefficient between the suitable habitat area and the water volume, and some proportional relationships were assumed in the calculation of the upper and lower bound values of ecological service value per unit area and net carbon sink capacity. These limitations would lead to systematic errors and limited usability of the model. Therefore, further investigation regarding the restoration and improvement of the suitable habitat area and the carrying capacity for rare migratory water birds is warranted.

## Figures and Tables

**Table 1:** Optimal water supply targets and the ecological water supplement optimization schemes under three scenarios (104 m^3^/year).

Intakes	Optimal Water Supply Targets	Local and Normal Water Supply			Flood Resource Supply		
		Low Flow Level	Medium Flow Level	High Flow Level	Low Flow Level	Medium Flow Level	High Flow Level
BIA	11,190.23	(6993.40, 8100.51)	(6993.40, 7066.28)	(6376.80, 6993.40)	(2158.38, 2413.19)	(2697.98, 3447.41)	(3372.47, 4136.90)
ZPS	7181.38	(3470.05, 4353.25)	(3470.05, 4353.25)	(3470.05, 4332.47)	(1450.66, 1621.91)	(1813.32, 2317.02)	(2266.65, 2780.42)
SIG	5709.35	(2524.10, 3190.80)	(2524.10, 3049.80)	(2521.49, 2524.10)	(1653.84, 1849.09)	(2067.30, 2641.55)	(2584.13, 3169.86)
HPS	843.85	(375.65, 489.90)	(375.65, 489.90)	(375.65, 446.41)	(207.36, 231.84)	(259.20, 331.20)	(324.00, 397.44)
Total	24,924.81	(13,363.20, 16,134.45)	(13,363.20, 14,959.23)	(13,363.20, 13,677.17)	(5470.24, 6116.03)	(6837.80, 8737.18)	(8547.24, 10,484.62)

Note: BIA = third branch channel of the Baishatan irrigation region; ZPS = Zhushan pumping station; SIG = Shijiangfang intake gate; and HPS = Haernao pumping station.

**Table 2:** Comparison of actual optimal water supply between interval-parameter fuzzy two-stage stochastic programming (IPFTSP) and interval-parameter two-stage stochastic programming model (IPTSP) models under different flood flow scenarios (10^4^ m^3^).

Flood Flow Scenario	Scenario Probability (%)	IPFTSP Model	IPTSP Model	Reduced Scale of Decision Space
Low Flow Level	50.00	(18,833.44, 22,250.48)	(18,833.44, 23,452.23)	26.02%
Medium Flow Level	38.00	(20,201.00, 23,696.41)	(20,201.00, 25,321.47)	31.74%
High Flow Level	12.00	(21,910.44, 24,161.79)	(21,910.44, 26,379.43)	49.62%

**Table 3:** Suitable habitat area for rare migratory water birds recovered prior to and after the optimization of the ecological water supplement schemes (10^3^ ha).

Regions	Habitat Area of Recommended Scheme	Habitat Area of Optimization Schemes		
		Low Flow Level	Medium Flow Level	High Flow Level
r = 1	6.22	(8.47, 10.17)	(8.81, 10.17)	(9.09, 10.17)
r = 2	5.95	(4.41, 6.17)	(4.85, 7.13)	(5.40, 7.60)
r = 3	0.79	(0.84, 1.06)	(0.88, 1.14)	(0.94, 1.14)
r = 4	0.40	(0.34, 0.47)	(0.38, 0.51)	(0.40, 0.51)
Total	13.36	(14.06, 17.88)	(14.92, 18.96)	(15.83, 19.43)
Target Value	13.36	(14.60, 18.47)		
Fuzzy Membership		(0.01, 0.83)		

**Table 4:** The recovered habitat area in the IPFTSP and IPTSP models under different flood flow scenarios (10^3^ ha).

Flood Flow Scenario	Scenario Probability (%)	Habitat Area in IPFTSP Model	Habitat Area in IPTSP Model	Reduced Scale of Decision Space
Low Flow Level	50.00	(14.06, 17.88)	(13.99, 18.59)	17.15%
Medium Flow Level	38.00	(14.92, 18.96)	(14.85, 19.78)	17.98%
High Flow Level	12.00	(15.83, 19.43)	(15.80, 20.53)	23.86%
Target value		(14.60, 18.47)	(14.53, 19.28)	18.28%

**Table 5:** The area of ecological function region recovered before and after the optimization of the ecological water supplement scheme (10^3^ ha).

Ecological Function Regions	Area of Ecological Function Region by Recommended Scheme	Area of Ecological Function Region by Optimal Schemes		
		Low Flow Level	Medium Flow Level	High Flow Level
Fish Pond	3.29	(2.63, 3.15)	(2.63, 3.78)	(3.46, 3.78)
Crab Pond	6.53	(5.50, 8.60)	(7.86, 8.73)	(8.65, 8.73)
Reed Wetland	7.44	(7.59, 8.62)	(7.01, 8.94)	(7.88, 8.95)
Marsh Wetland	16.98	(13.58, 17.86)	(13.81, 19.37)	(13.58, 20.31)
Total Area	34.23	(29.31, 38.23)	(31.31, 40.82)	(33.57, 41.78)

**Table 6:** Total recovered area of the ecological function region in the IPFTSP and IPTSP models under different flood flow scenarios (10^3^ ha).

Flood Flow Scenario	Scenario Probability (%)	Total Recovered Area in IPFTSP Model	Total Recovered Area in IPTSP Model	Interval Width Reduction Ratio
Low Flow Level	50.00	(29.31, 38.23)	(29.35, 41.01)	23.52%
Medium Flow Level	38.00	(31.31, 40.82)	(31.21, 44.14)	26.48%
High Flow Level	12.00	(33.57, 41.78)	(33.88, 46.04)	32.54%

**Table 7:** Ecological service value and net carbon sink generated by the ecological function regions recovered through multiple water supplement schemes under different flood flow scenarios.

	Recommended Scheme	IPFTSP Model	IPTSP Model	Interval Width Reduction Ratio
Ecological Service Value (in 10^8^ CNY)				
Low Flow Level	29.23	(25.07, 39.51)	(25.22, 41.89)	13.39%
Medium Flow Level		(27.18, 42.09)	(27.01, 44.89)	16.59%
High Flow Level		(29.31, 42.99)	(29.25, 46.76)	21.86%
Net Carbon Sink (in 10^4^ t)				
Low Flow Level	3.80	(3.34, 5.19)	(3.12, 5.33)	16.41%
Medium Flow Level		(3.14, 5.42)	(3.37, 6.34)	23.14%
High Flow Level		(3.45, 5.48)	(3.77, 6.60)	28.08%
